# Novel Yeasts Producing High Levels of Conjugated Linoleic Acid and Organic Acids in Fermented Doughs

**DOI:** 10.3390/foods10092087

**Published:** 2021-09-03

**Authors:** Michela Palla, Giuseppe Conte, Arianna Grassi, Semih Esin, Andrea Serra, Marcello Mele, Manuela Giovannetti, Monica Agnolucci

**Affiliations:** 1 Department of Agriculture, Food and Environment, University of Pisa, Via del Borghetto 80, 56124 Pisa, Italy; michela.palla@for.unipi.it (M.P.); giuseppe.conte@unipi.it (G.C.); arianna.grassi@agr.unipi.it (A.G.); andrea.serra@unipi.it (A.S.); marcello.mele@unipi.it (M.M.); manuela.giovannetti@unipi.it (M.G.); 2 Interdepartmental Research Centre “Nutraceuticals and Food for Health”, University of Pisa, 56124 Pisa, Italy; 3 Department of Translational Research and New Technologies in Medicine and Surgery, University of Pisa, 56123 Pisa, Italy; semih.esin@med.unipi.it

**Keywords:** fermented foods, functional yeasts, bioactive compounds, *Saccharomyces cerevisiae*, probiotic activity, anti-inflammatory capacity, propionic acid

## Abstract

Traditional fermented foods are obtained by a complex consortium of autochthonous microorganisms producing a wide variety of bioactive compounds, thus representing a reservoir of strains with new functional properties. Here, doughs obtained using five different wholegrain flours were singly fermented with selected yeast strains, which were evaluated for their functional traits. Lactate, volatile fatty acids and conjugated linoleic acid isomers produced by fermented doughs were detected by HPLC, while dough anti-inflammatory capacity was measured on human peripheral blood mononuclear cells by flow cytometry. Yeast potential probiotic activity was assessed by evaluating their resistance to simulated gastric and intestinal fluids. For the first time we report evidence of yeast strains producing high levels of the conjugated linoleic acid (CLA) isomer CLA 10-12tc and propionic acid, which are known for their specific health benefits. Moreover, such yeast strains showed an anti-inflammatory capacity, as revealed by a significantly decreased production of the strongly pro-inflammatory cytokine IL-1β. All our *Saccharomyces cerevisiae* strains were remarkably resistant to simulated gastric and intestinal fluids, as compared to the commercial probiotic strain. The two strains *S. cerevisiae* IMA D18Y and L10Y showed the best survival percentage. Our novel yeast strains may be exploited as valuable functional starters for the industrial production of cereal-based innovative and health-promoting fermented foods.

## 1. Introduction

Fermented foods have been part of the human diet since ancient times, representing one of the oldest ways to preserve foods. Over the last years, a renewed popularity of fermented foods has been observed worldwide, largely driven by their health benefits [1]. It has been estimated that more than 5000 different fermented products are currently produced and consumed worldwide [2], such as bread, fermented milks, kombucha, kimchi, sauerkraut etc. Fermented foods are defined as “foods made through desired microbial growth and enzymatic conversions of food components” [3]. They are produced through a fermentation process, in which a complex consortium of bacteria, yeasts and molds, originating from raw materials and processing environments (spontaneous fermentation) or added as starter cultures (controlled fermentation), promotes the breakdown of fermentable carbohydrates into simpler end-products [1]. Moreover, the spontaneous fermentation by autochthonous microorganisms leads to a wide variety of secondary metabolites, enhancing the bioavailability of nutrients, degrading toxic components and anti-nutritive factors, producing antioxidant and antimicrobial compounds, and bioactive compounds, such as folate, carotenoids, γ-aminobutyric acid (GABA), conjugated linoleic acid (CLA), and also organic acids, peptides, oligosaccharides and free polyphenols [4,5].

Among bioactive compounds, short-chain fatty acids (SCFA), are a group of organic acids characterized by small monocarboxylic acids with different chain lengths, ranging from two to six carbon atoms. Such compounds, that are generally known to affect the flavor and to inhibit the growth of spoilage microbes and pathogens in fermented products, have gained increasing attention, due to their proven positive effects on human health [6]. It is well known that SCFA, mainly acetic, propionic and butyric acid, may display immunomodulatory, antimicrobial and cholesterol lowering properties [7]. Many studies focused on SCFA production by gut microbiota, while limited literature is available on their production in fermented foods, although it is commonly known that, during fermentation, SCFA levels increase, compared with those of raw materials [6]. Actually, fruit [8,9,10], vegetables [11], tea [12,13,14], cereals [9,15,16,17,18,19] and mainly milk [20,21,22] have shown increases in SCFA level, when fermented by different bacterial and yeast strains.

Conjugated linoleic acid is a group of positional and geometric isomers of linoleic acid (c9,c12 octa-deca-dienoic acid) having conjugated double bonds. CLA has drawn the attention of scientists in the past few decades, because of its isomer-specific health-promoting properties. Recent studies have revealed that each isomer can have different effects on metabolism and cell functions: for example, CLA 9-11ct, also denominated rumenic acid, has anticarcinogenic biological activity in Caco-2 and MCF-10A cells, whereas CLA 10-12tc appears to be specifically responsible for the enhancement of energy metabolism in Chang liver cells and in the reduction of liver lipid content in Zucker rats [23]. There are currently limited reports of bioactivities for CLA isomers other than 9-11ct and 10-12tc. Among them, CLA 9-11tt or a mix of trans, trans CLA isomers showed anticancer and anti-inflammatory properties, antiplatelet aggregation, and hypocholesterolemic effects, and prevented fatty liver [24]. Numerous CLA isomers naturally occurs in a variety of foods, especially in those derived from ruminant animals (meat and dairy products), since they are formed during ruminal biohydrogenation by two important microorganisms, namely *Butyrivibrio fibrisolvens* and *Megasphaera elsdenii* [25], but they have been found also in some vegetables and seafood [26,27]. The predominant are CLA 9-11ct, accounting for approximately 90% of CLA intake in the diet [28], along with CLA 10-12tc and CLA 7-9tc, which are also present in minor but significant amounts. Besides ruminal microorganisms, several strains of food-grade bacteria, belonging to bifidobacteria, lactic acid bacteria, and propionibacteria, have been reported to produce CLA under in vitro conditions [29,30,31]. However, the few studies on microbial conversion of linoleic acid in food fermentations mainly investigated CLA-producing bacteria in dairy products [30,32,33,34], fermented pickle brines [35] and sourdough [36]. To the best of our knowledge, only Ando et al. [37] evaluated the ability to produce CLAs by fungi, screening more than 550 fungal strains and finding that the strain *Delacroixia coronate* IFO 8586 (Institute for Fermentation Osaka, Japan) was a potent producer of CLA.

Fermented foods and beverages may contain microbial strains with probiotic functions. Such strains are able to survive into gastro-intestinal tracts, as they are characterized by cell surface hydrophobicity and auto-aggregation, ability to grow at low pH and tolerate bile [38]. When probiotics are administrated in adequate amounts, they confer beneficial health effects to the host, enhancing human immune functions, improving digestion and nutrient assimilation. Most of the recognized probiotic microorganisms belong to lactic acid bacteria and Bifidobacteria, even if some yeast strains, belonging to *Saccharomyces cerevisiae* var. *boulardii*, *Kluyveromyces*, *Debaryomyces*, *Candida*, *Pichia*, *Torulaspora, Hanseniaspora* and *Metschnikowia* have recently been shown to possess probiotic properties [39]. *S. cerevisiae* var. *boulardii* CNCM I-745 (Collection Nationale de Cultures de Microorganismes, Institut Pasteur, Paris, France) was isolated from lychee fruit and currently is the only human probiotic yeast available on the market recommended for the prevention and treatment of human gastrointestinal diseases, the control of serum cholesterol and acute diarrhea in adults and children. It shows also positive effects in the treatment of *Clostridium difficile* and *Helicobacter pylori* infections [40]. Potential probiotic strains of *Kluyveromyces* were isolated from cheese [38,41] and kefir [42], *S. cerevisiae* from Brazilian beverage caxiri [43], kefir [42], wine [40] and sourdough [44], while *Pichia kluyveri* and *Pichia kudriavzevii* strains were isolated from fermented cocoa [42] and cereal-based Nigerian traditional fermented food [45].

Traditional fermented foods and beverages represent a reservoir of strains with potential novel functional properties, to be used as functional starters. In this view, the screening and selection of multifunctional strains are of great interest in the food industry [46], also considering that such microorganisms are classified as GRAS (generally recognized as safe) [47]. So far, most of the studies on fermented foods have focused on functional bacterial strains, although a few works reported that yeasts may increase the content of bioactive compounds responsible for specific health benefits [48,49].

In a previous work, 39 yeast strains, isolated from cereal-based fermented foods and beverages, were characterized for their pro-technological and functional traits, when individually used to ferment five different wholegrain flours, and the two strains with the highest leavening, phytase and antioxidant activities were detected for each flour [50]. Here, doughs obtained using five different wholegrain flours were fermented with the two selected strains and evaluated for their ability to produce bioactive compounds, i.e., organic acids and CLA and for their anti-inflammatory capacity. The potential role of yeast strains as probiotics was also assessed.

## 2. Materials and Methods

### 2.1. Microorganisms

Nine *S. cerevisiae* and one *Kazachstania humilis* isolates utilized here, belonged to our collection in the Department of Agriculture, Food and Environment (DAFE) of the University of Pisa (Table 1), and were isolated from sourdoughs [51]. They were selected for their leavening ability, phytase and antioxidant activities, when singly inoculated in doughs obtained using five different wholegrain flours [50]. Along with such isolates, two commercial yeasts and two reference strains were used (Table 1).

All yeasts were routinely cultivated on Yeast Peptone Dextrose agar [YEPD, 1% yeast extract (Oxoid, Milan, Italy), 1% bacteriological peptone (Oxoid, Milan, Italy), 2% dextrose (Sigma-Aldrich, Milan, Italy), 2% agar (Sigma-Aldrich, Milan, Italy)] and incubated at 25 °C for 48 h.

### 2.2. Phylogenetic Relationship among Yeast Isolates

The phylogenetic relationship among the nine *S. cerevisiae* isolates, along with *K. humilis* IMA G23Y, the commercial baker’s yeast *S. cerevisiae* Zeus IBA, the commercial probiotic *S. cerevisiae* var. *boulardii* CNCM I-745 and the reference strains was assessed by inter-delta region analysis. Amplification reactions were performed using δ1 (5′-CAA AAT TCA CCT ATA/TTC TCA-3′) and δ2 (5′-GTG GAT TTT TAT TCC AAC A-3′) primers (Eurofins Genomics, Ebersberg, Germany) and 160 ng of DNA, as reported in Palla et al. [52]. All gels were visualized by UV and captured as TIFF format files by the UVI 1D v. 16.11a program for FIRE READER V4 gel documentation systems (Uvitec Cambridge, Eppendorf, Milan, Italy). Inter-delta profiles were digitally processed and analyzed with the BioNumerics software version 7.6 (Applied Maths, St-Martens-Latem, Belgium). Profiles were compared using the band matching tool with a position tolerance and optimization of 0.5%, and similarity was calculated using the Dice’s coefficient. For cluster analysis, unpaired group method with arithmetic average (UPGMA) trees with highest resampling support, in a permutation sample of size 200, were constructed. The reproducibility of fingerprints was assessed.

### 2.3. In Vitro Production of Organic Acids 

Our yeasts were in vitro characterized for their ability to produce organic acids. Ten microliters of the yeast liquid cultures grown overnight, at 25 °C on Wallerstein Laboratory Nutrient (WLN) (Oxoid, Basingstoke, UK) broth medium, corresponding to 10^6^ cfu/mL, were inoculated on the same medium supplemented with 2% agar and 0.03 g/L methylene blue (Sigma-Aldrich, Milan, Italy). Plates were then incubated at 25 °C for 24 h and visually evaluated for the presence of *halo zones* around colonies. In particular, the production of organic acids was calculated as the difference between the total diameter (colony + *halo zone*) and the colony diameter. Indeed, WLN media contains bromocresol green, which is a dye used as pH indicator in growth medium; decreases of pH change the color of the media from blue to yellow, as the result of organic acid production.

### 2.4. Dough Preparation

Doughs were prepared using five different flours supplied by the Department of Agricultural Forest and Food Sciences, University of Torino, Italy. *S. cerevisiae* IMA D20Y and D22Y, *S. cerevisiae* IMA L22Y and L17Y, *S. cerevisiae* IMA D8Y and L6Y, *S. cerevisiae* IMA L22Y and D18Y, *S. cerevisiae* IMA L15Y and L10Y were singly inoculated in doughs obtained from three wheat one winter emmer and a hull-less spring barley varieties as described in Palla et al. [50].

Doughs were prepared in triplicate, as described in Palla et al. [50]. Briefly, 100 g of dough with a dough yield of 160 (dough yield = dough weight × 100/flour weight), supplemented with chloramphenicol (0.1 g/L), were mixed manually for 5 min and fermented at 30 °C for 24 h. For the inoculum, each yeast was cultivated into YEPD broth at 25 °C overnight; cells were then harvested at 10,000× *g*, 10 min, 4 °C, washed in sterile saline-peptone water (0.9% NaCl, 0.1% bacteriological peptone) and re-suspended in tap water at the cell density of ca. 6.0–7.0 Log cfu/mL. Not inoculated doughs, supplemented with chloramphenicol (0.1 g/L) and cycloheximide (0.1 g/L), were used as controls. Doughs singly inoculated with *Kazachstania humilis* IMA G23Y and with Zeus IBA commercial baker’s yeast were also prepared using all the flours.

### 2.5. In Vivo Detection of Organic Acids

The analysis of lactate and volatile fatty acids (VFA) (2:0, acetic; 3:0, propionic; 4:0, butyric) of dough samples was performed by HPLC according to the procedure described by Sandri et al. [53] with some modifications. Briefly, 15 g of dough were diluted with 70 mL of 0.1 N H_2_SO_4_ aqueous solution and homogenized for 2 min by UltraTurrax (IKA^®^-Werke GmbH & Co. KG, Staufen, Germany). After incubation at room temperature, the mix was centrifuged (5000× *g* for 15 min at 4 °C) to separate the liquid phase from the solid residuals and the liquid phase subsequently microfiltered (SLMV033RS, 0.45-μm Millex-HV, Merck-Millipore, Billerica, MA, USA). The resulting sample was directly injected in the HPLC apparatus using an Aminex 85 HPX-87 H ion exclusion column (300 mm × 7.8 mm; 9-μm particle size; Bio-Rad, Milan, Italy) kept at 40 °C; the detection wavelength was 220 nm. The analyses were carried out applying an isocratic elution (flux 0.6 mL/min) with a 0.008 N H_2_SO_4_ solution as mobile phase; the injection loop was 20 μL. Individual SCFA and lactic acid were identified using a standard solution of 4.50 mg/mL of lactic acid, 5.40 mg/mL of acetic acid, 5.76 mg/mL of propionic acid, 7.02 mg/mL of butyric acid in 0.1 N H_2_SO_4_ (69,775, 338,826, 402,907, 58,360, respectively; Sigma-Aldrich, Milano Italy). Quantification was achieved using an external calibration curve based on the standards described above.

### 2.6. In Vivo Detection of Fatty Acid and CLA Isomers

Total lipids of dough were extracted with a chloroform/methanol solution (2:1, *v*/*v*), according to [54]. Fatty acids methyl esters (FAME) were prepared using a combined basic and acid procedure according to [55] with some modifications. Briefly, 1 mg of C19:0 (internal standard) was added to 20 mg of lipids and the derivatization was performed using a sodium methoxide 0.5M solution in methanol followed by hydrochloric acid in methanol (1:1). FAME were separated and identified using a GC-FID (GC 2000 plus, Shimadzu, Columbia, MD, United States) according to Mele et al. [56].

Conjugated Linoleic Acid isomers were separated and quantified by three silver ion HPLC columns (ChromSpher 5 Lipids, Varian, Middelburg, Netherlands; 250mm, 4.6mm i.d.) according to Conte et al. [57]. Briefly, CLA isomers were eluted using a fresh mixture of acetonitrile 0.1% (*v*/*v*) in hexane at a flow of 1 mL/min. The injection loop was 20 μL, and UV detection was performed at a wavelength of 233 nm. Quantitative measurements were performed through a calibration curve, using a high purity individual c9, t11 and t10, c12 CLAs (Matreya Inc., Pleasant Gap, PA, USA). The CLA mix standard (Sigma Chemical Co., St. Louis, MO, USA), and published isomeric profiles [58] were also used to help identify the CLA isomers in meat TL. Total fatty acids were expressed as g/100 g of total lipids, whereas individual CLA were expressed as mg/100 g of the total lipids.

### 2.7. Ex Vivo Anti-Inflammatory Activity 

Water/salt-soluble extracts (WSE) of fermented doughs were prepared according to the method originally described by Osborne [59] and modified by Weiss et al. [60]. Samples of 1.6 g dough were suspended in 4 mL of Tris-HCl buffer (50 mM, pH 8.8), incubated for 1 h at 4 °C under stirring (150 rpm) and centrifuged at 10,000× *g* for 30 min. The supernatant was removed, filtered and stored at −20 °C.

The anti-inflammatory capacity of doughs was assessed on human Peripheral Blood Mononuclear Cells (PBMC) obtained from healthy donors attending the Transfusion center of Pisa University Hospital or from healthy volunteers. An informed consent was obtained from each donor. The study was conducted in accordance with the Declaration of Helsinki, and the protocol was approved by the local Ethical Committee (CEAVNO—Comitato Etico di Area Vasta Nord Ovest, Tuscany Region, Italy; Protocol 34743, 28/06/2018). PBMC were isolated by standard gradient protocol as described previously [61]. From each WSE 10 mg/mL (final concentration) was added to 4 × 10^5^ PBMC in the presence of 1 μg/mL *E. coli* O26:B6 lipopolysaccharide (LPS) (Sigma-Aldrich, St. Louis, MO, USA) and cultured for 24 h at 37 °C, 5% CO_2_. Culture conditions in which PBMC were incubated with 1 μg/mL *E. coli* LPS only or with the complete culture medium RPMI-1640 (unstimulated) were established as positive and negative controls, respectively. Following the incubation culture supernatants were collected from each condition, aliquoted and kept at −80 °C until use.

The levels of a panel of cytokines (IL-1β, IL-6, IL-10, and TNF- α) present in the co-culture supernatants were determinated by a flow cytometer based multi-bead capture assay (LEGENDplex^TM^ Multi-Analyte Flow Assay Kit, BioLegend Inc., San Diego, CA, USA) according to manufacturer’s instructions [62]. Sensitivities of the assay were as follows: IL-1β, 0.65 ± 0.47 pg/mL; IL-6, 0.97 ± 1.46 pg/mL; IL-10, 0.77 ± 1.18 pg/mL; TNF-α 0.88 ± 0.27 pg/mL. Samples were acquired in a BD Accuri C6 flow cytometer (BD Biosciences, San Jose, CA, USA), analyzed with LegendPlex v8.0 Software (BioLegend Inc., San Diego, CA, USA) and referred to a standard curve. Results were expressed as pg/mL.

### 2.8. Resistance to Simulated Gastric and Intestinal Fluids of Yeast Isolates

*S. cerevisiae* isolates were in vitro characterized for their ability to resist to simulated gastric and intestinal fluids, as described in Palla et al. [51]. The commercial probiotic *S. cerevisiae* var. *boulardii* CNCM I-745, isolated from Codex (CODEX, Zambon Italia S.r.l., Bresso, Italy), was used as the positive control strain.

Exponentially phase grown cells were harvested, washed and suspended as described in Zárate et al. [63]. The final pH was adjusted to 2.0, 3.0 and 8.0. The suspension was incubated at 37 °C under anaerobic conditions and agitation (180 rpm) to simulate peristalsis. Aliquots of this suspension were taken at 0, 90 and 180 min, and viable counts were determined on YEPD agar. The effect of gastric digestion was also determined by suspending cells in 10% (*w/v*) Reconstituted Skimmed Milk—RSM (Sigma-Aldrich, Milan, Italy), before inoculation of simulated gastric juice at pH 2.0. The final pH after the addition of RSM was ca. 3.0. 

After 180 min of gastric digestion, cells were harvested at 8000× *g* for 15 min at 4 °C and suspended in simulated intestinal fluid which contained 0.1% (*w/v*) pancreatin (Sigma–Aldrich Co.) and 0.15% (*w/v*) Ox-bile salt (Sigma–Aldrich Co.) at pH 8.0. The suspension was incubated at 37 °C under anaerobic conditions and agitation (180 rpm) and aliquots were taken at 90 and 180 min, and viable counts were determined on YEPD agar.

The analyses were performed in triplicate and the survival percentage was calculated as (Log cfu/mL (T_360_)/Log cfu/mL (T_0_)) × 100 where T_360_ is the yeast concentration after 360 min of incubation and T_0_ is the initial inoculum.

### 2.9. Statistical Analysis

Statistical analyses of in vivo organic acids, fatty acid and CLA isomers data were carried out by the following linear model, using JMP software (SAS Institute Inc., Cary, NC, USA):

y_ij_ = μ + Y_i_ + ε_ij_

where y_ij_ = dependent variables; m = mean; Y_i_ = fixed effect of the i^th^ strain (5 levels); ε_ij_ = random residual. Since the same strains were not used for each of the flours, the model was applied considering each flour separately and evaluating the effect of the respective strains. Least-square means with their standard errors were reported, and treatment effects were declared significant at *p* < 0.05. Post-hoc analysis was performed by a Tukey test.

For statistical analyses of ex vivo anti-inflammatory activity one-way ANOVA followed by Tukey-Kramer multiple comparisons test was used. A *p*-value of <0.05 was considered significant.

## 3. Results

### 3.1. Phylogenetic Relationship among Yeast Strains

**Figure 1:**
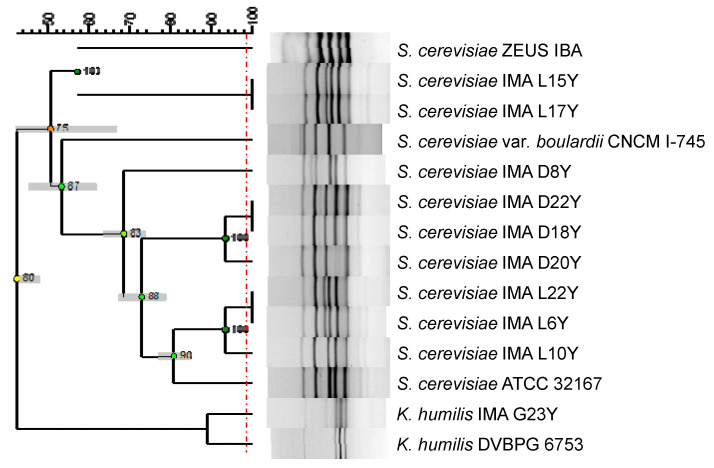
Dendrogram obtained from unpaired group method with arithmetic average (UPGMA) analysis, using Dice’s coefficient, based on inter-delta profiles of the 10 yeast strains tested, along with the commercial baker’s yeast Zeus IBA, the commercial probiotic *S. cerevisiae* var. *boulardii* CNCM I-745 (Collection Nationale de Cultures de Microorganismes, Institut Pasteur, Paris, France) and the two reference strains. The red line indicates the similarity value (98.7%) for separation of biotypes. Standard deviation is shown at each node by a grey bar. Cophenetic correlation is shown at each branch by numbers and coloured dots, ranging between green-yellow-orange-red, according to decreasing values.

The phylogenetic relationship among *S. cerevisiae* strains, *K. humilis* IMA G23Y, the commercial baker’s yeast *S. cerevisiae* Zeus IBA, the commercial probiotic *S. cerevisiae* var. *boulardii* CNCM I-745 and two reference strains (Table 1) was evaluated by inter-delta regions analysis. The dendrogram (Figure 1), obtained comparing the inter-delta profiles, grouped all the *S. cerevisiae* strains in a main cluster with 51% of similarity. *K. humilis* strains branched separately at 44% similarity. Among *S. cerevisiae* isolates, two main clusters were identified: the first one grouped *S. cerevisiae* IMA L15Y and L17Y with *S. cerevisiae* Zeus IBA at a similarity of 57%. The second cluster grouped the other seven isolates with the commercial probiotic strain *S. cerevisiae* var. *boulardii* CNCM I-745, with 54% of similarity.

### 3.2. In Vitro Production of Organic Acids

To functionally characterize the yeast isolates, a preliminary screening was carried out by plate assay, in order to test their ability to produce organic acids. All our isolates showed a *halo zone*, revealing the production of organic acids, which ranged from 4.00 ± 0.33 mm (*S. cerevisiae* IMA L6Y) to 5.33 ± 0.33 mm (*S. cerevisiae* IMA L10Y) (Table S1). Therefore, all isolates were further investigated by quantitative screenings.

### 3.3. In Vivo Detection of Organic Acids

**Figure 2:**
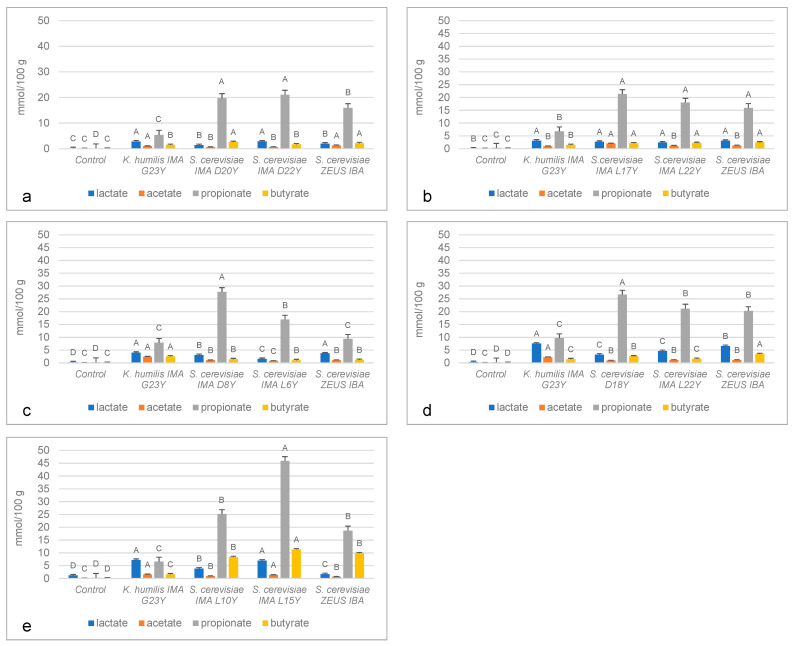
Histogram showing the organic acid profiles of fermented doughs individually inoculated with the yeast strains, obtained using (**a**) conventional red-grained wheat variety *T. aestivum* L. cv Aubusson, (**b**) yellow-grained wheat variety *T. aestivum* L. cv Bona Vita, (**c**) blue-grained wheat variety *T. aestivum* L. cv Skorpion, (**d**) winter emmer variety *Triticum turgidum* subsp. *dicoccum* var. *Schrank*, Giovanni Paolo and (**e**) hull-less spring barley variety *Hordeum vulgare* L. var. *nudum Hook*, Rondo. A–D: means within organic acid with different letters significantly differ (*p* ≤ 0.01).

Yeast isolates were singly inoculated in doughs obtained using the appropriate flour, fermented at 30 °C for 24 h and analyzed for their ability to produce organic acids, such as lactic, acetic, propionic and butyric acid, by HPLC. Overall, all the fermented doughs showed a significantly higher content of the four acids than not inoculated doughs, where the production was almost absent (Figure 2 and Tables S2–S6). Among organic acids, propionic acid was the most abundant one. In particular, in fermented doughs inoculated with *S. cerevisiae* strains, propionic acid content ranged from 9.39 ± 2.44 mmol/100 g of dough in the blue-grained cv. Skorpion doughs inoculated with *S. cerevisiae* Zeus IBA (Figure 2c and Table S2) to 45.85 ± 2.31 mmol/100 g of dough in barley doughs inoculated with *S. cerevisiae* IMA L15Y (Figure 2e and Table S3). On the contrary, fermented doughs inoculated with the strain *K. humilis* IMA G23Y showed lower propionic acid contents, which ranged from 5.45 ± 2.75 to 9.60 ± 2.09 mmol/100 g of dough in conventional red-grained common wheat (Figure 2a and Table S4) and in emmer doughs (Figure 2d and Table S5), respectively. In the doughs inoculated with such a strain, lactic acid production resulted higher than in those obtained using *S. cerevisiae* strains. In particular, the highest lactate content was found in emmer (7.56 ± 0.13 mmol/100 g of dough) and in barley (7.27 ± 0.28 mmol/100 g of dough) doughs (Figure 2d and Table S5, Figure 2e and Table S3, respectively).

### 3.4. In Vivo Detection of Fatty Acid and Cla Isomers

**Figure 3:**
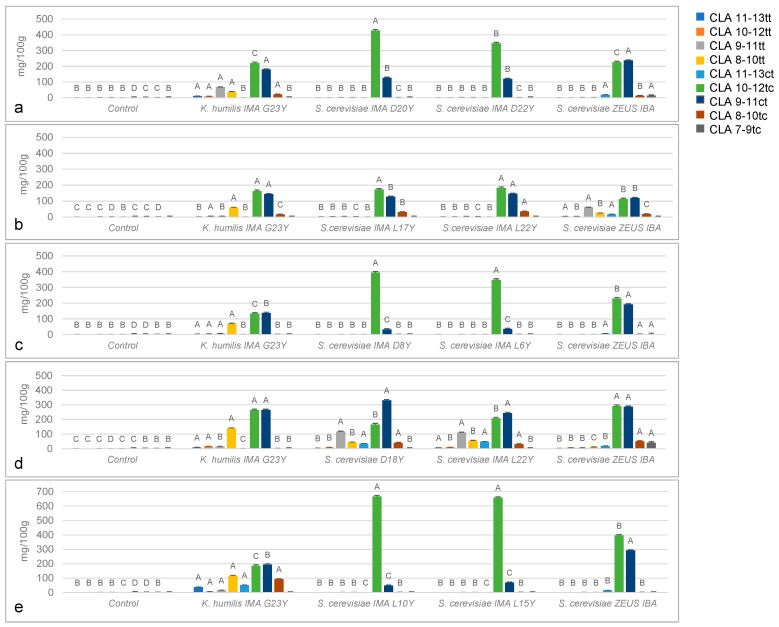
Histogram showing conjugated linoleic acid (CLA) (mg/100 g of total lipids) profiles of fermented doughs individually inoculated with the yeast strains, obtained using (**a**) conventional red-grained wheat variety *T. aestivum* L. cv Aubusson, (**b**) yellow-grained wheat variety *T. aestivum* L. cv Bona Vita, (**c**) blue-grained wheat variety *T. aestivum* L. cv Skorpion, (**d**) winter emmer variety *Triticum turgidum* subsp. *dicoccum* var. *Schrank*, Giovanni Paolo and (**e**) hull-less spring barley variety *Hordeum vulgare* L. var. *nudum Hook*, Rondo. A–D: means within CLA isomers with different letters significantly differ (*p* ≤ 0.01).

Fatty acid composition and CLA isomers profiles demonstrated a significant effect of yeast strains on their production (Figure 3 and Tables S2–S6). Overall, all the fermented doughs showed the presence of CLA isomers, as opposed to controls, where these acids were not detected (Figure 3). The CLA level was on average 567 mg/100 g of total lipids, ranging from 340 mg/100 g of total lipids in yellow-grained wheat doughs to 801 mg/100 g of total lipids in emmer doughs. The total amount of CLA did not show significant differences among the strains in the same doughs. Different profiles were detected depending on the strain: *S. cerevisiae* mainly synthesized CLA 10-12tc and CLA 9-11ct, while *K. humilis* was characterized by a lower production of these isomers and a greater synthesis of trans-trans isomers (in particular CLA 8-10tt). Among *S. cerevisiae*, Zeus IBA, IMA L17Y, L22Y and D18Y strains yielded the same amount of CLA 10-12tc and CLA 9-11ct, while IMA D20Y, D22Y, D8Y, L6Y, L10Y and L15Y strains synthesized about 4 times higher CLA 10-12tc amounts than CLA 9-11ct.

Our results highlighted another important effect of yeasts on the nutraceutical profile of the doughs. As shown in Tables S2–S6, fermented doughs showed a significant increase (approx. +32%) in the level of omega-3 polyunsaturated fatty acids. In addition, *S. cerevisiae* strains accumulated a higher content of such acids than *K. humilis* IMA G23Y, which showed higher values than the control only in yellow-grained wheat. No significant differences were detected in fermented doughs obtained with emmer flour.

### 3.5. Ex Vivo Anti-Inflammatory Activity

**Figure 4:**
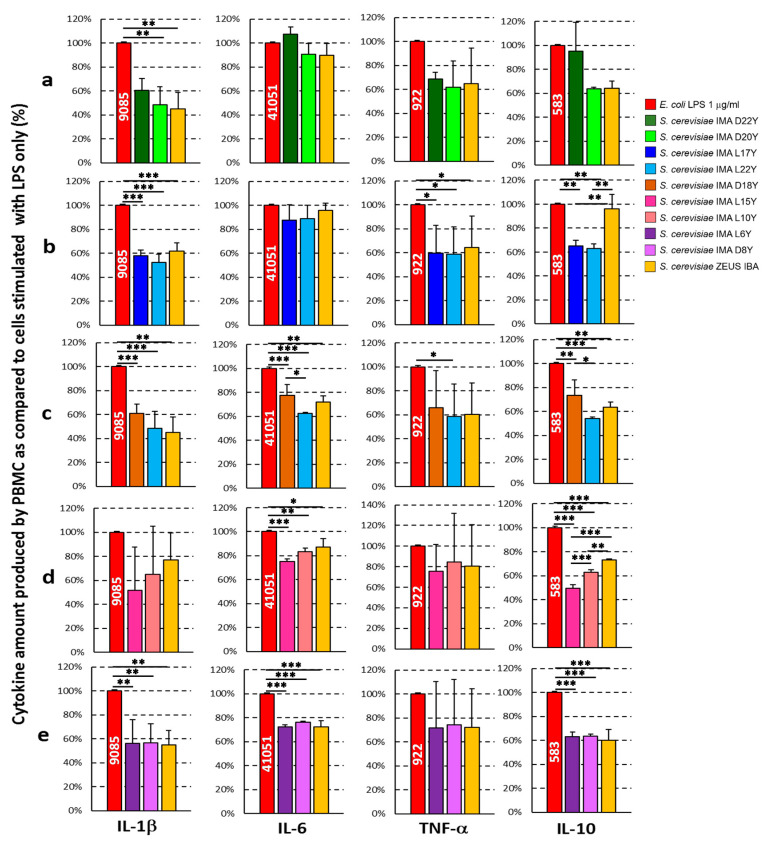
Ex vivo cytokine production profiles of peripheral blood mononuclear cells (PBMC) incubated 24 h with 1 μg/mL *E. coli* LPS in the presence of water/salt soluble extracts (WSE) obtained from fermented doughs individually inoculated with the yeast strains. The quantity of each cytokine produced was depicted as percent of the cytokine produced by PBMC stimulated only with LPS in the absence of WSE. The numbers on LPS histograms (red bars) depict mean cytokine amount (pg/mL) produced by LPS stimulated PBMC. (**a**) conventional red-grained wheat variety *T. aestivum* L. cv Aubusson, (**b**) yellow-grained wheat variety *T. aestivum* L. cv Bona Vita, (**c**) winter emmer variety *Triticum turgidum* subsp. *dicoccum* var. *Schrank*, Giovanni Paolo, (**d**) hull-less spring barley variety *Hordeum vulgare* L. var. *nudum Hook*, Rondo, and (**e**) blue-grained wheat variety *T. aestivum* L. cv Skorpion. Data are reported as mean ± SD of three different experiments. * *p* < 0.05, ** *p* < 0.01, *** *p* < 0.001, one-way ANOVA followed by Tukey-Kramer multiple comparisons test.

To study the anti-inflammatory capacity of fermented doughs obtained by inoculating the five different flours with the selected yeast strains, as well as with a commercial baker’s yeast, PBMC obtained from healthy blood donors were stimulated ex vivo with 1 μg/mL *E. coli* LPS for 24 h in the presence of water/salt soluble dough extracts. Pro-inflammatory (IL-1β, IL-6, TNF-α) and regulatory/anti-inflammatory (IL-10) cytokines were quantified in the culture supernatants and compared to those obtained in the culture conditions where PBMC were stimulated with LPS only. A vast majority of the extracts demonstrated the ability to decrease the cytokine production from LPS stimulated PBMC for most of the cytokines tested (Figure 4). In particular, the production of IL-1β, a strong pro-inflammatory cytokine mainly produced by monocytes upon LPS stimulation, was significantly decreased by ca. 50% in almost all yeast strain-dough combinations (Figure 4). 

An anti-inflammatory activity for WSEs was also present for TNF-α and IL-6 secretion, albeit to a lesser extent. In addition, the production of IL-10, a regulatory/anti-inflammatory cytokine, was decreased as well for most of the yeast strain–flour combinations (Figure 4). The WSE obtained from doughs inoculated with commercial baker’s yeast showed an anti-inflammatory ability slightly less effective as compared to other WSEs.

### 3.6. Resistance to Simulated Gastric and Intestinal Fluids of Yeast Isolates

**Figure 5:**
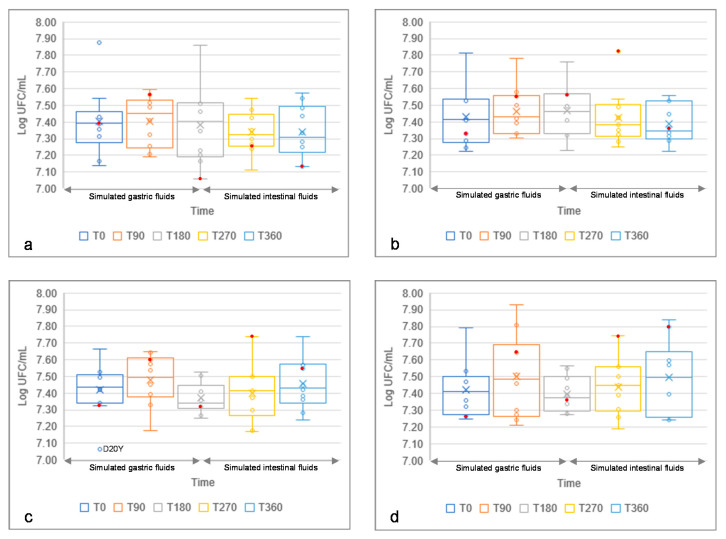
Boxplot showing the survival (Log cfu/mL) of 10 *Saccharomyces cerevisiae* strains under simulated gastric conditions (0–180 min) at pH 2.0 (**a**), 3.0 (**b**), 8.0 (**c**) and 2.0 with added reconstituted skim milk (10%) (**d**), and further intestinal digestion (180–360 min) at pH 8.0. Circles indicate the isolates; the red ones indicate the commercial probiotics’ yeast from Codex. Median values are reported (×). The top and the bottom of the box represent the 75th and 25th percentile of the data, respectively. The top and the bottom of the bars represent the 5th and the 95th percentile of the data, respectively.

The resistance of yeasts to simulated gastric and intestinal conditions is a pre-requisite to characterize strains as probiotics, based on their potential immunomodulatory effects [64]. *S. cerevisiae* isolates, along with the commercial probiotic *S. cerevisiae* var. *boulardii* CNCM I-745, used as the positive control, were incubated under simulated gastric and intestinal conditions. Different pHs were used to assess the influence of the simulated gastric juice on yeasts survival, in order to rule out the effect of low pH [65]. Moreover, to simulate the effect of the food matrix during gastric transit [63], yeast isolates were also incubated in simulated gastric juice added with reconstituted skimmed milk. Overall, all our yeast strains did not show significant decreases of cell counts in any condition at any time (cell counts ranged from 7.07 ± 0.03 to 7.93 ± 0.02 Log cfu/mL), showing a marked resistance when incubated under simulated gastric and intestinal conditions (Figure 5). Such results were consistent with those of the commercial probiotic strain (Figure 5). Compared to the initial viability, *S. cerevisiae* IMA L10Y and D18Y showed a survival percentage higher than 100% in all the tested conditions (Figure S1). 

## 4. Discussions

In this work, for the first time, we characterized novel yeast strains for their ability to produce high levels of the conjugated linoleic acid isomer CLA 10-12tc and of propionic acid, when singly inoculated in doughs obtained from five different wholegrain cereal flours. Moreover, an anti-inflammatory capacity of such strain/dough combinations was revealed by a significantly decreased production of the strongly pro-inflammatory cytokine IL-1β.

During fermentation, sourdough yeasts metabolize carbohydrates mostly into ethanol and CO_2_, producing also other compounds, such as aldehydes, alcohols, esters, ketones and organic acids, which affect the aroma of baked goods [66,67]. Recently, the discovery of short-chain fatty acids beneficial effects on human health led researchers to investigate sourdough microbiota producing high levels of SCFA, in order to obtain breads with enhanced nutritional and nutraceutical properties. Here, our selected yeasts singly inoculated in different wholegrain flours were able to produce organic acids, mainly propionic acid. To the best of our knowledge, this is the first evidence of high-level production of propionic acid by *S. cerevisiae* strains in fermented doughs. Propionic acid is usually accumulated in the colon, as a common end-product of dietary carbohydrates, resistant starches and dietary fibers fermentation by gut microbiota, or can be produced directly by probiotic LAB [8]. Propionate exerts immunosuppressive actions, increase satiety, regulate fat synthesis and cholesterol in the liver, and improves tissue insulin sensitivity preventing obesity and type 2 diabetes [68,69]. Naturally, propionic acid occurs in low quantities in some foods, such as milk, showing anti-fungal and anti-bacterial properties. Many studies showed that propionic acid content of raw materials increased after fermentation, mostly after propionibacterial fermentation [69]. Alvarez-Martin et al. [20] found that propionic acid was produced during milk fermentation by several yeast strains belonging to the species *Candida famata*, *Debaryomyces hansenii*, *Kluyveromyces lactis*, *Pichia fermentans*, *Pichia membranifaciens* and *Yarrowia lipolytica*. Conversely, in doughs fermented by *S. cerevisiae* the main organic acids detected were acetic and butyric acids [70], and succinic acid [17,18]. Further studies will determine the fate of the high levels of propionic acid found in our doughs after baking, although no safety concerns have been detected when utilized as food additive [71].

In our study, the highest content of propionic acid was found in barley doughs fermented with different *S. cerevisiae* strains, and in such doughs also butyric acid was produced, highlighting that the biochemical composition of the flour, along with the microbial species, affect organic acid production. Additionally, butyric acid may have beneficial impact on human health, improving insulin sensitivity, protecting against diet-induced obesity and colon cancer [8,69]. Barley have recently gained increasing interest in the food industry, due to its high β-glucan content, which represents a major component of soluble fiber implicated in hypocholesterolemia, hypoglycemia and in reducing incidence of chemically induced colon cancer in experimental animals.

Another important group of functional metabolites is CLA isomers, which showed nutraceutical properties for human health. CLA is abundant in products of animal origin, especially those deriving from ruminants, but they occur also in plant oils and selected seafoods. CLA content of food originating from unprocessed raw materials can change as a result of microbial fermentation [33], nevertheless, only few reports investigated CLA-producing bacteria in fermented foods. Among them, only Black et al. [36], studying the antifungal activity of some lactobacilli, found that *Levilactobacillus* *hammesii* was able to convert linoleic acid into hydroxy fatty acids, during sourdough fermentation. The reason why some bacteria convert linoleic acid to CLA is still unclear. As linoleic acid has been shown to be toxic to many bacteria, Jiang et al. [72] supposed that the production of CLA may be a detoxification mechanism for bacterial cells.

For the first time, our study showed that our novel yeast strains were able to produce CLA isomers in singly fermented cereal doughs. *S. cerevisiae* strains produced almost exclusively CLA9-11ct and CLA 10-12tc, while *K. humilis* strain synthesized a smaller quantity of these fatty acids, but a higher content of isomers trans-trans. Considering such results, we can suppose a different pathway of isomerization of linoleic acid between these two yeast genera. Unfortunately, few data are available about yeast’s CLA synthesis process. Only Ando et al. [37], investigating the CLA metabolic pathway among 550 fungi, found that *Delacroixia coronata* IFO 8586 was able to produce CLA9-11ct from trans-vaccenic acid, through fatty acid Δ9-desaturation reaction. Moreover, in our results, different CLA isomers profiles were obtained fermenting each of the five flours with the same yeast strain (*S. cerevisiae* Zeus IBA or *K. humilis* IMA G23Y), highlighting the effect of the substrate composition. Such results are consistent with those obtained in our previous work, where we in vivo characterized 39 yeast strains for their leavening ability, phytase activity and polyphenols content using the same five different wholegrain flours, finding that such functional traits were strongly affected by the type of flour used [50]. Moreover, as for propionic acid production, the highest production of CLA 10-12tc was detected in barley doughs fermented with the selected *S. cerevisiae* strains, highlighting that the composition of such flour could possess high levels of precursors promoting the production of these two functional compounds. CLA 10-12tc is directly involved in the control of body fat accumulation, by the regulation of lipid oxidation pathway [73]. CLA 9-11ct showed positive effect on immune and inflammatory responses, including reduction of adverse effects of immune challenges, reduction of colonic inflammation, decreased antigen-induced cytokine production in immune-competent cells, reduction of allergic type immune responses, and modulation of the production of cytokines, prostaglandins, and leukotrienes [73]. Regarding the other CLA isomers, only few reports investigated the bioactivities of trans, trans CLA isomers, reporting anticancer, anti-inflammation, antiplatelet aggregation, and hypocholesterolemic effects [24].

Interestingly, the analyses of the cytokine production pattern of PBMC stimulated with LPS in the presence of yeast strain/dough combinations used in the present study demonstrated an anti-inflammatory effect. This effect was evident by the diminished production of the three pro-inflammatory cytokines analyzed (i.e., IL-1β, IL-6 and TNF-α) from PBMC cultured in the presence of WSEs as compared to PBMC stimulated only with LPS. In addition, the observed decrease in the amount of IL-10, a regulatory/anti-inflammatory cytokine, could indicate an ability of the yeast/dough combinations to interfere with LPS stimulatory effect [74].

Formation of CLA and other functional metabolites can be accomplished during food fermentation, but this process could also be amplified by probiotic microorganisms directly interacting with the human host. Consequently, our *S. cerevisiae* strains were evaluated for their ability to resist to simulated gastric and intestinal conditions, which is a pre-requisite for the selection of probiotics. All our *S. cerevisiae* strains, just as the commercial probiotic *S. cerevisiae* var. *boulardii* CNCM I-745, showed a remarkable survival, growing at 37 °C temperature, with 0.15% *w*/*v* bile salts, at pH 2.0 and up to pH 8.0. In particular, two strains (*S. cerevisiae* IMA D18Y and L10Y) showed the best survival percentage in all the tested conditions. Interestingly, such two strains branched in the same inter-delta cluster of the commercial probiotic strain *S. cerevisiae* var. *boulardii* CNCM I-745. Consistently with our findings, Perricone et al. [44], characterizing yeast strains isolated from Altamura sourdough for their probiotic traits, found promising *S. cerevisiae* strains with high survival percentage under simulated gastro-intestinal conditions. As the sourdough system is a stressful environment, characterized by a low pH, as well as the digestive system, sourdough-associated yeasts are well adapted to such peculiar conditions, explaining the high survival percentage found among our yeasts under simulated gastro-intestinal conditions. Although the most common probiotic strains are bacteria, generally belonging to lactic acid bacteria and Bifidobacteria [43], recent studies focused also on yeast strains with probiotic potential from sourdough and other fermented foods, and the list of potential probiotic yeasts is continuously updated [38,75,76,77]. Yeasts possess several beneficial properties and advantages in comparison to bacteria, because they have better cell adherence, intrinsic resistance to antibiotics, and did not show, so far, any transfer of genetic material to gut bacteria [46]. Moreover, yeast strains belonging to *S. cerevisiae* are considered safe microbes, widely distributed in fermented foods and beverages and used safely since ancient times [42]. In bread production, the major limitation for probiotic applications is the inactivation of microorganisms during the baking step. Sourdough-associated microorganisms with functional and probiotic traits could be selected as probiotic starter cultures for cereal-based food or beverages with improved healthy and sensory features. Indeed, the use of yeasts to ferment plant matrices in substitution to milk was recently proposed in order to obtain non-dairy beverages as an alternative to the traditional dairy products for vegans and lactose-intolerant consumers [78]. In such a way, human health may be improved, consuming foods containing multi-functional probiotics as part of the daily diet [46].

It is interesting to note that the two *S. cerevisiae* strains, IMA D18Y and L10Y, showing the best survival percentage to simulated gastro-intestinal conditions, possessed also a high phytase activity, when inoculated in wholegrain flours [50]. Such a trait allows the hydrolyzation of phytate, an abundant anti-nutritional factor in cereal flours which chelates minerals such as iron and zinc, depleting their bioavailability and uptake. Since phytate is the predominant form of phosphorus in cereal grains, and humans are unable to produce phytase, the probiotic potential of our *S. cerevisiae* strains should be deeply investigated, in order to use such strains as multi-functional probiotic starters to combat mineral deficiency [79].

## Figures and Tables

**Table 1:** Yeast isolates and reference strains used in this study.

Strains ^a^	Source of Isolation
*Saccharomyces cerevisiae* IMA D8Y, D18Y, D20Y, D22Y, L6Y, L10Y, L15Y, L17Y, L22Y, *Kazachstania humilis* IMA G23Y	Tuscan wheat sourdoughs [51]
*Saccharomyces cerevisiae* Zeus IBA	Commercial baker’s yeast of Zeus IBA srl
*Saccharomyces cerevisiae* var. *boulardii* CNCM I-745	Commercial probiotics’ yeast from Codex (CODEX, Zambon Italia S.r.l., Bresso, Italy)
*Saccharomyces cerevisiae* ATCC 32167	Unknown
*Kazachstania humilis* DBVPG 6753	San Francisco Sourdough Bread

^a^ IMA = International Microbial Archives, Department of Agriculture, Food and Environment, University of Pisa, Pisa, Italy; CNCM = Collection Nationale de Cultures de Microorganismes, Institut Pasteur, Paris, France; ATCC = American Type culture Collection, Manassas, Virginia, USA; DBVPG = International Collection of Department of Agricultural, Food and Environmental Science, University of Perugia, Perugia, Italy.

## Data Availability

The datasets analyzed during the current study are available from the corresponding author on reasonable request.

## Supplementary Materials

The following are available online at https://www.mdpi.com/article/10.3390/foods10092087/s1, **Table S1**: In vitro yeasts’ production of organic acids. **Table S2**: Fatty acid (g/100 g of total lipids), organic acid (mmol/100 g of dough) and conjugated linoleic acid (CLA) (mg/100 g of total lipids) profiles of blue-grained wheat variety *T. aestivum* L. cv Skorpion fermented doughs, individually inoculated with the two selected yeast strains (*S. cerevisiae* D8Y and L6Y), *K. humilis* IMA G23Y and the commercial baker’s yeast *S. cerevisiae* Zeus IBA. **Table S3**: Fatty acid (g/100 g of total lipids), organic acid (mmol/100 g of dough) and conjugated linoleic acid (CLA) (mg/100 g of total lipids) profiles of hull-less spring barley variety *Hordeum vulgare* L. var. *nudum Hook*, Rondo fermented doughs, individually inoculated with the two selected yeast strains (*S. cerevisiae* L10Y and L15Y), *K. humilis* IMA G23Y and the commercial baker’s yeast *S. cerevisiae* Zeus IBA. **Table S4**: Fatty acid (g/100 g of total lipids), organic acid (mmol/100 g of dough) and conjugated linoleic acid (CLA) (mg/100 g of total lipids) profiles of conventional red-grained wheat variety *T. aestivum* L. cv Aubusson fermented doughs, individually inoculated with the two selected yeast strains (*S. cerevisiae* D20Y and D22Y), *K. humilis* IMA G23Y and the commercial baker’s yeast *S. cerevisiae* Zeus IBA. **Table S5**: Fatty acid (g/100 g of total lipids), organic acid (mmol/100 g of dough) and conjugated linoleic acid (CLA) (mg/100 g of total lipids) profiles of winter emmer variety *Triticum turgidum* subsp. *dicoccum* var. *Schrank*, Giovanni Paolo fermented doughs, individually inoculated with the two selected yeast strains (*S. cerevisiae* D18Y and L22Y), *K. humilis* IMA G23Y and the commercial baker’s yeast *S. cerevisiae* Zeus IBA. **Table S6**: Fatty acid (g/100 g of total lipids), organic acid (mmol/100 g of dough) and conjugated linoleic acid (CLA) (mg/100 g of total lipids) profiles of yellow-grained wheat variety *T. aestivum* L. cv Bona Vita fermented doughs, individually inoculated with the two selected yeast strains (*S. cerevisiae* L17Y and L22Y), *K. humilis* IMA G23Y and the commercial baker’s yeast *S. cerevisiae* Zeus IBA. **Figure S1**: Survival percentage of the 10 *Saccharomyces cerevisiae* strains after incubation under simulated gastric conditions at pH 2.0 (**a**), 3.0 (**b**), 8.0 (**c**), and 2.0, with reconstituted skim milk added (10%) (**d**), for 180 min, and further intestinal digestion at pH 8.0 for 360 min. Stars are the mean values (*n* = 3).
