# Supplementary material for: Novel Yeasts Producing High Levels of Conjugated Linoleic Acid and Organic Acids in Fermented Doughs
Source: Foods. 2021 Sep 3;10(9):2087. doi: 10.3390/foods10092087 (PMC8466363; doi:10.3390/foods10092087)
Supplement: Supplementary file 1 [file foods-10-02087-s001.zip › foods-1330723-supplementary.pdf]

Novel yeasts producing high levels of conjugated linoleic acid and organic acids in fermented doughs

Michela Palla, Giuseppe Conte, Arianna Grassi, Semih Esin, Andrea Serra, Marcello Mele, Manuela Giovannetti, Monica Agnolucci

Supplementary Table S1. *In vitro* yeasts' production of organic acids

Strains[a]	Halo zone (mm)	SE[1]
Saccharomyces cerevisiae IMA L10Y	5.33	0.33
Kazachstania humilis IMA G23Y	5.00	0.29
Saccharomyces cerevisiae IMA L15Y	5.00	0.00
Saccharomyces cerevisiae IMA L22Y	5.00	0.33
Kazachstania humilis DBVPG 6753	4.83	0.50
Saccharomyces cerevisiae ATCC 32167	4.67	0.17
Saccharomyces cerevisiae IMA D20Y	4.67	0.33
Saccharomyces cerevisiae IMA L17Y	4.67	0.58
Saccharomyces cerevisiae IMA D18Y	4.33	0.33
Saccharomyces cerevisiae IMA D22Y	4.33	0.33
Saccharomyces cerevisiae IMA D8Y	4.33	0.33
Saccharomyces cerevisiae IMA L6Y	4.00	0.33
Saccharomyces cerevisiae Zeus IBA	4.00	0.67

[a]IMA=International Microbial Archives, Department of Agriculture, Food and Environment, University of Pisa, Pisa, Italy; ATCC=American Type culture Collection, Manassas, Virginia, USA; DBVPG=International Collection of Department of Agricultural, Food and Environmental Science, University of Perugia, Perugia, Italy.
[1]SE: Standard Error.

Supplementary Table S2. Fatty acid (g/100 g of total lipids), organic acid (mmol/100 g of dough) and conjugated linoleic acid (CLA) (mg/100 g of total lipids) profiles of blue-grained wheat variety *T. aestivum* L. cv Skorpion fermented doughs, individually inoculated with the two selected yeast strains (*S. cerevisiae* D8Y and L6Y), *K. humilis* IMA G23Y and the commercial baker's yeast *S. cerevisiae* Zeus IBA

	-	*K. humilis* IMA G23Y	*S.cerevisiae* IMA D8Y	*S.cerevisiae* IMA L6Y	*S. cerevisiae* Zeus IBA	SE[1]	P-value
Saturated	*12.60*	*13.82*	*15.23*	*14.31*	*14.86*	*0.93*	*0.368*
C14:0	0.08	0.08	0.10	0.11	0.08	0.02	0.598
C15:0	0.08	0.08	0.08	0.09	0.09	0.01	0.882
C16:0	11.06	12.35	13.10	12.44	13.14	0.86	0.469
C17:0	0.09	0.09	0.10	0.10	0.10	0.01	0.507
C18:0iso	0.00^{B}	0.06^{A}	0.06^{A}	0.04^{A}	0.10^{A}	0.01	<0.001
C18:0	0.85	0.78	1.23	0.92	0.82	0.18	0.445
C20:0	0.12	0.12	0.22	0.19	0.11	0.04	0.238
C22:0	0.16	0.18	0.18	0.18	0.21	0.03	0.756
C23:0	0.12	0.05	0.07	0.14	0.15	0.03	0.112
Monounsaturated	*8.51*	*10.04*	*11.84*	*10.06*	*11.17*	*0.72*	*0.067*
C16:1c9	0.09^{b}	0.14^{a}	0.19^{a}	0.14^{a}	0.16^{a}	0.02	0.029
C17:1c9	0.05	0.04	0.05	0.05	0.05	0.01	0.143
C18:1c9	7.78	9.01	10.65	9.02	9.92	0.66	0.094
C18:1c11	0.61	0.76	0.85	0.75	0.85	0.05	0.052
Polyunsaturated	*35.27*	*40.31*	*39.07*	*36.54*	*43.23*	*2.53*	*0.262*
C18:2n-6	31.59	35.70	33.49	31.54	37.31	2.19	0.317
C18:3n-3	1.89	2.22	2.31	1.88	2.29	0.14	0.135
C20:2n-6	0.08^{b}	0.07^{b}	0.09^{b}	0.13^{a}	0.06^{b}	0.02	0.015
C20:4n-6	0.04^{B}	0.09^{A}	0.12^{A}	0.10^{A}	0.11^{A}	0.01	0.008
C20:5n-3	0.60	0.74	1.16	1.02	1.18	0.16	0.093
C22:3n-3	0.69^{b}	0.57^{b}	1.13^{a}	1.11^{a}	1.26^{a}	0.14	0.019
C22:4n-6	0.06^{B}	0.05^{B}	0.00^{C}	0.00^{C}	0.27^{A}	0.03	<0.001
C22:5n-6	0.00^{C}	0.06^{B}	0.05^{B}	0.14^{A}	0.04^{B}	0.02	0.004
C22:5n-3	0.15^{a}	0.09^{a}	0.02^{c}	0.00^{c}	0.01^{c}	0.03	0.022
C22:6n-3	0.16	0.25	0.26	0.21	0.26	0.03	0.171
CLA[2] isomers	*0.00^{C}*	*396.71^{A}*	*450.67^{A}*	*348.06^{B}*	*433.80^{A}*	*28.38*	*<0.001*
CLA_11-13tt	0.00^{B}	2.73^{A}	0.00^{B}	0.00^{B}	0.00^{B}	0.14	<0.001
CLA_10-12tt	0.00^{B}	4.98^{A}	0.00^{B}	0.00^{B}	0.00^{B}	0.20	<0.001
CLA_9-11tt	0.00^{B}	6.05^{A}	0.00^{B}	0.00^{B}	0.00^{B}	0.18	<0.001
CLA_8-10tt	0.00^{B}	70.84^{A}	0.00^{B}	0.00^{B}	0.00^{B}	1.52	<0.001
CLA_11-13ct	0.00^{B}	0.00^{B}	0.00^{B}	0.00^{B}	7.24^{A}	1.00	0.001
CLA_10-12tc	0.00^{D}	133.84^{C}	394.54^{A}	348.94^{A}	229.86^{B}	23.65	<0.001
CLA_9-11ct	0.00^{D}	138.19^{B}	33.96^{C}	35.53^{C}	191.80^{A}	5.51	<0.001
CLA_8-10tc	0.00^{B}	0.00^{B}	0.00^{B}	0.00^{B}	1.29^{A}	0.57	<0.001
CLA_7-9tc	0.00^{B}	0.00^{B}	0.00^{B}	0.00^{B}	2.95^{A}	2.10	
Index							
ω-6	31.78	35.97	33.75	31.91	37.79	2.19	0.296
ω-3	3.50^{b}	3.87^{b}	4.87^{a}	4.22^{a}	5.00^{a}	0.36	0.040
ω-6/ω-3	9.11^{A}	9.30^{A}	6.99^{B}	7.56^{B}	7.60^{B}	0.35	0.003
Organic acid	*0.534^{C}*	*16.711^{B}*	*33.216^{A}*	*20.263^{B}*	*15.339^{B}*	*3.140*	*<0.001*
lactate	0.250^{D}	3.981^{A}	3.074^{B}	1.557^{C}	3.700^{A}	0.259	<0.001
acetate	0.000^{C}	2.398^{A}	1.013^{B}	0.746^{C}	1.052^{B}	0.684	<0.001
propionate	0.284^{D}	7.833^{C}	27.684^{A}	16.901^{B}	9.391^{C}	2.442	<0.001
butyrate	0.000^{C}	2.498^{A}	1.446^{B}	1.059^{B}	1.196^{B}	0.654	<0.001

[1]SE: Standard Error; [2]CLA: Conjugated Linoleic Acid.

$^{a-c}$: means within rows with different letters significantly differ ($P \leq 0.05$)

$^{A-D}$: means within rows with different letters significantly differ ($P \leq 0.01$)

Supplementary Table S3. Fatty acid (g/100 g of total lipids), organic acid (mmol/100 g of dough) and conjugated linoleic acid (CLA) (mg/100 g of total lipids) profiles of hull-less spring barley variety *Hordeum vulgare* L. var. *nudum Hook*, Rondo fermented doughs, individually inoculated with the two selected yeast strains (*S. cerevisiae* L10Y and L15Y), *K. humilis* IMA G23Y and the commercial baker's yeast *S. cerevisiae* Zeus IBA

	-	*K. humilis* IMA G23Y	*S.cerevisiae* IMA L10Y	*S.cerevisiae* IMA L15Y	*S. cerevisiae* Zeus IBA	SE[1]	P-value
Saturated	*16.74*^{C}	*18.95*^{A}	*19.58*^{A}	*20.27*^{A}	*18.06*^{B}	*0.54*	*0.007*
C14:0	0.16	0.24	0.20	0.19	0.19	0.02	0.251
C15:0	0.08	0.09	0.08	0.09	0.08	0.00	0.079
C16:0	14.68^{D}	16.51^{B}	16.58^{B}	17.53^{A}	15.62^{C}	0.39	0.004
C17:0	0.07	0.08	0.09	0.09	0.08	0.00	0.053
C18:0iso	0.00^{B}	0.15^{A}	0.00^{B}	0.00^{B}	0.00^{B}	0.01	<0.001
C18:0	0.99	1.27	1.42	1.22	1.40	0.17	0.456
C20:0	0.17^{C}	0.20^{C}	0.65^{A}	0.43^{B}	0.19^{C}	0.04	<0.001
C22:0	0.22	0.26	0.23	0.26	0.22	0.02	0.160
C23:0	0.10^{b}	0.04^{b}	0.20^{a}	0.25^{a}	0.22^{a}	0.04	0.011
Monounsaturated	*11.18*^{b}	*13.10*^{a}	*12.78*^{a}	*12.31*^{a}	*13.72*^{a}	*0.48*	*0.036*
C16:1c9	0.09^{c}	0.15^{b}	0.20^{a}	0.21^{a}	0.23^{a}	0.02	0.016
C17:1c9	0.13	0.13	0.16	0.15	0.15	0.01	0.082
C18:1c9	11.21	12.11	11.56	11.06	12.45	0.46	0.142
C18:1c11	0.59	0.68	0.68	0.69	0.70	0.02	0.132
Polyunsaturated	*33.43*	*36.31*	*32.98*	*34.81*	*30.89*	*1.56*	*0.226*
C18:2n-6	28.78	30.80	28.12	28.52	24.53	1.56	0.153
C18:3n-3	1.89	2.06	1.92	1.80	1.78	0.18	0.803
C20:2n-6	0.06^{B}	0.07^{B}	0.18^{A}	0.23^{A}	0.07^{B}	0.03	0.003
C20:4n-6	0.08^{C}	0.20^{A}	0.127^{B}	0.20^{A}	0.13^{B}	0.01	<0.001
C20:5n-3	1.05^{B}	1.13^{B}	0.11^{C}	1.23^{B}	1.52^{A}	0.09	<0.001
C22:3n-3	1.30^{B}	0.87^{C}	1.51^{B}	1.81^{A}	1.91^{A}	0.16	0.006
C22:4n-6	0.13^{A}	0.06^{B}	0.00^{C}	0.00^{C}	0.13^{A}	0.01	<0.001
C22:5n-6	0.03^{B}	0.06^{A}	0.03^{B}	0.00^{C}	0.00^{C}	0.00	<0.001
C22:5n-3	0.00^{B}	0.14^{A}	0.00^{B}	0.00^{B}	0.00^{B}	0.00	<0.001
C22:6n-3	0.11	0.16	0.12	0.13	0.12	0.02	0.434
CLA[2] isomers	*0.00*^{B}	*710.43*^{A}	*715.04*^{A}	*728.50*^{A}	*703.85*^{A}	*10.38*	*<0.001*
CLA_11-13tt	0.00^{B}	37.80^{A}	0.00^{B}	0.00^{B}	0.00^{B}	8.53	<0.001
CLA_10-12tt	0.00^{B}	5.89^{A}	0.00^{B}	0.00^{B}	0.00^{B}	0.17	<0.001
CLA_9-11tt	0.00^{B}	15.07^{A}	0.00^{B}	0.00^{B}	0.00^{B}	1.25	<0.001
CLA_8-10tt	0.00^{B}	117.57^{A}	0.00^{B}	0.00^{B}	0.00^{B}	2.68	<0.001
CLA_11-13ct	0.00^{C}	52.22^{A}	0.00^{C}	0.00^{C}	15.00^{B}	1.93	<0.001
CLA_10-12tc	0.00^{D}	186.31^{C}	667.41^{A}	658.85^{A}	396.09^{B}	8.99	<0.001
CLA_9-11ct	0.00^{D}	194.81^{B}	48.32^{C}	69.39^{C}	292.83^{A}	13.43	<0.001
CLA_8-10tc	0.00^{B}	92.74^{A}	0.00^{B}	0.00^{B}	0.00^{B}	5.78	<0.001
Index							
ω-6	29.09	31.19	28.46	28.95	24.86	1.57	0.152
ω-3	4.35^{B}	4.35^{B}	3.67^{C}	4.98^{A}	5.33^{A}	0.11	<0.001
ω-6/ω-3	6.69^{B}	7.19^{A}	7.74^{A}	5.83^{C}	4.67^{D}	0.39	0.002
Organic acid	*1.515*^{D}	*17.096*^{C}	*38.326*^{B}	*65.610*^{A}	*30.980*^{B}	*3.043*	*<0.001*
lactate	1.216^{D}	7.267^{A}	3.867^{B}	7.038^{A}	1.709^{C}	0.278	0.023
acetate	0.026^{C}	1.575^{A}	0.909^{B}	1.365^{A}	0.655^{B}	0.278	0.034
propionate	0.273^{D}	6.637^{C}	25.172^{B}	45.855^{A}	18.735^{B}	2.314	<0.001
butyrate	0.000^{D}	1.617^{C}	8.377^{B}	11.351^{A}	9.880^{B}	0.204	<0.001

[1]SE: Standard Error; [2]CLA: Conjugated Linoleic Acid.

$^{a\text{-}c}$: means within rows with different letters significantly differ ($P \leq 0.05$)

$^{A\text{-}D}$: means within rows with different letters significantly differ ($P \leq 0.01$)

Supplementary Table S4. Fatty acid (g/100 g of total lipids), organic acid (mmol/100 g of dough) and conjugated linoleic acid (CLA) (mg/100 g of total lipids) profiles of conventional red-grained wheat variety *T. aestivum* L. cv Aubusson fermented doughs, individually inoculated with the two selected yeast strains (*S. cerevisiae* D20Y and D22Y), *K. humilis* IMA G23Y and the commercial baker's yeast *S. cerevisiae* Zeus IBA

	-	*K. humilis* IMA G23Y	*S. cerevisiae* IMA D20Y	*S. cerevisiae* IMA D22Y	*S. cerevisiae* Zeus IBA	SE[1]	P-value
Saturated	*14.35*	*15.67*	*14.92*	*14.72*	*14.32*	*0.79*	*0.231*
C14:0	0.10	0.09	0.10	0.10	0.08	0.01	0.143
C15:0	0.09	0.08	0.10	0.10	0.09	0.01	0.266
C16:0	12.64	14.23	13.04	12.78	12.53	0.46	0.135
C17:0	0.10	0.09	0.07	0.09	0.09	0.09	0.766
C18:0iso	0.01^{C}	0.00^{C}	0.06^{A}	0.03^{B}	0.05^{A}	0.00	<0.001
C18:0	0.94	0.94	1.09	1.09	0.87	0.06	0.078
C20:0	0.13^{c}	0.14^{b}	0.18^{b}	0.21^{a}	0.16^{b}	0.02	0.031
C22:0	0.17^{B}	0.10^{C}	0.21^{A}	0.18^{AB}	0.20^{A}	0.02	0.008
C23:0	0.11^{B}	0.00^{D}	0.03^{D}	0.07^{C}	0.20^{A}	0.01	<0.001
Monounsaturated	*9.63*	*10.86*	*11.37*	*10.92*	*10.72*	*0.41*	*0.110*
C16:1c9	0.09^{B}	0.16^{A}	0.16^{A}	0.15^{A}	0.17^{A}	0.01	0.009
C17:1c9	0.01	0.04	0.03	0.04	0.04	0.01	0.091
C18:1c9	8.86	9.91	10.29	9.94	9.60	0.39	0.177
C18:1c11	0.62	0.73	0.72	0.69	0.68	0.03	0.097
Polyunsaturated	*39.88^{b}*	*46.58^{a}*	*40.29^{b}*	*38.80^{b}*	*38.19^{b}*	*1.67*	*0.033*
C18:2n-6	35.88^{b}	42.08^{a}	34.71^{b}	34.64^{b}	32.54^{b}	1.64	0.019
C18:3n-3	2.12^{B}	2.56^{A}	1.97^{B}	2.00^{B}	1.82^{B}	0.12	0.011
C20:2n-6	0.17^{B}	0.24^{A}	0.07^{C}	0.09^{C}	0.06^{C}	0.01	<0.001
C20:4n-6	0.09	0.08	0.11	0.11	0.10	0.01	0.407
C20:5n-3	0.59^{B}	0.32^{B}	1.05^{A}	0.11^{C}	1.27^{A}	0.12	<0.001
C22:3n-3	0.67^{C}	0.26^{D}	1.20^{A}	0.96^{B}	1.36^{A}	0.12	<0.001
C22:4n-6	0.06^{C}	0.31^{A}	0.09^{B}	0.03^{C}	0.12^{B}	0.02	<0.001
C22:5n-6	0.09^{A}	0.00^{C}	0.05^{B}	0.05^{B}	0.04^{B}	0.00	<0.001
C22:5n-3	0.00^{C}	0.07^{B}	0.15^{A}	0.01^{C}	0.02^{C}	0.04	<0.001
C22:6n-3	0.20^{B}	0.12^{C}	0.27^{A}	0.23^{B}	0.25^{A}	0.02	0.002
CLA[2] isomers	*0.00^{B}*	*548.15^{A}*	*555.23^{A}*	*535.56^{A}*	*560.35^{A}*	*23.87*	*<0.001*
CLA_11-13tt	0.00^{B}	10.71^{A}	0.00^{B}	0.00^{B}	0.00^{B}	1.99	<0.001
CLA_10-12tt	0.00^{B}	8.98^{A}	0.00^{B}	0.00^{B}	0.00^{B}	0.35	<0.001
CLA_9-11tt	0.00^{B}	68.23^{A}	0.00^{B}	0.00^{B}	0.00^{B}	0.38	<0.001
CLA_8-10tt	0.00^{B}	38.08^{A}	0.00^{B}	0.00^{B}	0.00^{B}	1.47	<0.001
CLA_11-13ct	0.00^{B}	0.00^{B}	0.00^{B}	0.00^{B}	18.51^{A}	1.41	<0.001
CLA_10-12tc	0.00^{D}	221.25^{C}	427.89^{A}	346.72^{B}	225.25^{C}	28.54	<0.001
CLA_9-11ct	0.00^{C}	178.95^{A}	126.93^{B}	119.72^{B}	236.64^{A}	30.29	<0.001
CLA_8-10tc	0.00^{C}	21.57^{A}	0.00^{C}	0.00^{C}	12.10^{B}	3.22	0.002
CLA_7-9tc	0.00^{B}	0.00^{B}	0.00^{B}	0.00^{B}	13.23^{A}	0.93	<0.001
Index							
ω-6	36.29^{b}	42.71^{a}	35.03^{b}	34.91^{b}	32.87^{b}	1.62	0.014
ω-3	3.56^{B}	3.32^{B}	4.64^{A}	3.31^{B}	4.73^{A}	0.17	<0.001
ω-6/ ω-3	10.18^{B}	12.87^{A}	7.54^{C}	10.56^{B}	7.04^{C}	0.42	<0.001
Organic acid	*0.39^{C}*	*10.76^{B}*	*24.41^{A}*	*26.31^{A}*	*21.32^{A}*	*3.43*	*<0.001*
lactate	0.21^{C}	2.82^{A}	1.43^{B}	2.84^{A}	1.99^{B}	0.36	<0.001
acetate	0.02^{C}	1.01^{A}	0.61^{B}	0.65^{B}	1.30^{A}	0.14	<0.001
propionate	0.16^{D}	5.45^{C}	19.76^{A}	21.09^{A}	15.87^{B}	2.75	<0.001
butyrate	0.00^{C}	1.48^{B}	2.61^{A}	1.72^{B}	2.15^{A}	0.31	<0.001

[1]SE: Standard Error; [2]CLA: Conjugated Linoleic Acid.

[a-c]: means within rows with different letters significantly differ ($P \leq 0.05$)

[A-D]: means within rows with different letters significantly differ ($P \leq 0.01$)

Supplementary Table S5. Fatty acid (g/100 g of total lipids), organic acid (mmol/100 g of dough) and conjugated linoleic acid (CLA) (mg/100 g of total lipids) profiles of winter emmer variety *Triticum turgidum* subsp. *dicoccum* var. *Schrank*, Giovanni Paolo fermented doughs, individually inoculated with the two selected yeast strains (*S. cerevisiae* D18Y and L22Y), *K. humilis* IMA G23Y and the commercial baker's yeast *S. cerevisiae* Zeus IBA

	-	*K. humilis* IMA G23Y	*S. cerevisiae* D18Y	*S. cerevisiae* IMA L22Y	*S. cerevisiae* Zeus IBA	SE[1]	P-value
Saturated	20.39^{A}	13.95^{B}	14.011^{B}	15.33^{B}	13.10^{B}	*0.48*	*<0.001*
C14:0	0.07	0.09	0.07	0.14	0.06	0.01	0.348
C15:0	0.07	0.10	0.10	0.10	0.10	0.01	0.223
C16:0	12.82^{a}	12.07^{a}	12.14^{a}	13.02^{a}	11.31^{b}	0.28	0.011
C17:0	0.08	0.07	0.07	0.08	0.07	0.00	0.061
C18:0iso	0.00^{B}	0.03^{A}	0.04^{A}	0.04^{A}	0.03^{A}	0.01	0.009
C18:0	1.16	1.11	1.10	1.48	1.00	0.14	0.215
C20:0	0.17	0.19	0.18	0.19	0.18	0.00	0.056
C22:0	0.12^{B}	0.24^{A}	0.25^{A}	0.22^{A}	0.22^{A}	0.02	0.002
C23:0	0.00^{C}	0.05^{B}	0.01^{C}	0.01^{C}	0.07^{A}	0.01	<0.001
Monounsaturated	*16.40*	*16.05*	*15.95*	*16.04*	*15.63*	*0.40*	*0.749*
C16:1c9	0.18	0.18	0.19	0.19	0.18	0.01	0.844
C17:1c9	0.04	0.03	0.03	0.04	0.03	0.00	0.146
C18:1c9	15.35	14.92	14.78	14.80	14.53	0.38	0.660
C18:1c11	0.81	0.79	0.78	0.78	0.77	0.81	0.738
Polyunsaturated	45.30^{a}	43.01^{a}	38.90^{b}	39.19^{b}	*39.36b*	*1.50*	*0.044*
C18:2n-6	35.56	38.41	34.50	34.40	34.73	1.43	0.121
C18:3n-3	2.75	2.59	2.06	2.15	2.15	0.13	0.113
C20:2n-6	0.23^{A}	0.06^{B}	0.06^{B}	0.06^{B}	0.05^{B}	0.00	<0.001
C20:4n-6	0.03^{c}	0.13^{a}	0.04^{c}	0.05^{c}	0.08^{b}	0.02	0.019
C20:5n-3	0.17^{C}	0.39^{B}	0.67^{A}	0.53^{A}	0.68^{A}	0.08	0.003
C22:3n-3	0.12^{C}	0.29^{B}	0.44^{B}	0.37^{B}	0.63^{A}	0.07	0.006
C22:4n-6	0.26^{A}	0.06^{B}	0.07^{B}	0.07^{B}	0.06^{B}	0.01	<0.001
C22:5n-6	0.00^{C}	0.05^{A}	0.01^{B}	0.02^{B}	0.02^{B}	0.01	<0.001
C22:5n-3	0.08^{A}	0.05^{B}	0.02^{C}	0.02^{C}	0.02^{C}	0.01	<0.001
C22:6n-3	0.10	0.18	0.15	0.13	0.11	0.02	0.056
CLA[2] isomers	0.00^{B}	800.56^{A}	797.05^{A}	792.70^{A}	784.27^{A}	0.02	<0.001
CLA_11-13tt	0.00^{C}	8.77^{A}	3.53^{B}	6.23^{A}	2.23^{B}	1.30	0.006
CLA_10-12tt	0.00^{C}	15.71^{A}	9.23^{B}	9.77^{B}	6.88^{B}	1.52	<0.001
CLA_9-11tt	0.00^{C}	15.11^{B}	118.34^{A}	111.55^{A}	5.99^{B}	14.08	<0.001
CLA_8-10tt	0.00^{D}	139.94^{A}	43.90^{B}	55.80^{B}	10.27^{C}	8.14	<0.001
CLA_11-13ct	0.00^{C}	0.00^{C}	35.04^{A}	48.56^{A}	18.42^{B}	6.37	<0.001
CLA_10-12tc	0.00^{C}	263.82^{A}	165.66^{B}	207.69^{B}	292.16^{A}	26.36	<0.001
CLA_9-11ct	0.00^{B}	265.02^{A}	331.04^{A}	243.93^{A}	287.16^{A}	43.54	<0.001
CLA_8-10tc	0.00^{B}	0.00^{B}	40.95^{A}	30.67^{A}	51.39^{A}	9.75	0.011
CLA_7-9tc	0.00^{B}	0.00^{B}	0.00^{B}	0.00^{B}	41.21^{A}	0.57	
Index							
ω-6	42.09^{a}	38.71^{b}	34.69^{c}	35.15^{c}	34.93^{c}	1.44	0.017
ω-3	3.21	3.50	3.33	3.20	3.60	0.13	0.215
ω-6/ω-3	13.10^{A}	11.07^{B}	10.41^{B}	11.03^{B}	9.79^{B}	0.48	0.006
Organic acid	0.54^{C}	20.87^{B}	33.37^{A}	28.46^{A}	31.41^{A}	*2.48*	*<0.001*
lactate	0.32^{D}	7.56^{A}	3.24^{C}	4.64^{C}	6.60^{B}	0.13	<0.001
acetate	0.02^{C}	2.22^{A}	0.92^{B}	1.10^{B}	1.10^{B}	0.27	<0.001
propionate	0.20^{D}	9.60^{C}	26.63^{A}	21.18^{B}	20.25^{B}	2.09	<0.001
butyrate	0.00^{D}	1.44^{C}	2.59^{B}	1.545^{C}	3.47^{A}	0.21	<0.001

[1]SE: Standard Error; [2]CLA: Conjugated Linoleic Acid.

[a-c]: means within rows with different letters significantly differ ($P \leq 0.05$)

[A-D]: means within rows with different letters significantly differ ($P \leq 0.01$)

Supplementary Table S6. Fatty acid (g/100 g of total lipids), organic acid (mmol/100 g of dough) and conjugated linoleic acid (CLA) (mg/100 g of total lipids) profiles of yellow-grained wheat variety *T. aestivum* L. cv Bona Vita fermented doughs, individually inoculated with the two selected yeast strains (*S. cerevisiae* L17Y and L22Y), *K. humilis* IMA G23Y and the commercial baker's yeast *S. cerevisiae* Zeus IBA

	-	*K. humilis* IMA G23Y	*S.cerevisiae* IMA L17Y	*S.cerevisiae* IMA L22Y	*S. cerevisiae* Zeus IBA	SE[1]	P-value
Saturated	*22.39*^{A}	*13.52*^{B}	*12.48*^{B}	*12.35*^{B}	*13.15*^{B}	*0.45*	*<0.001*
C14:0	0.14	0.11	0.08	0.07	0.08	0.30	<0.001
C15:0	0.07	0.08	0.07	0.07	0.08	0.00	0.132
C16:0	12.25^{A}	11.79^{A}	10.63^{B}	10.63^{B}	11.51^{A}	0.21	<0.001
C17:0	0.08	0.08	0.08	0.08	0.08	0.00	0.094
C18:0iso	0.00^{C}	0.05^{A}	0.03^{B}	0.03^{B}	0.05^{A}	0.01	0.002
C18:0	1.02	1.04	0.91	0.90	0.94	0.05	0.172
C20:0	0.11	0.12	0.11	0.11	0.11	0.00	0.318
C22:0	0.05^{C}	0.20^{A}	0.15^{B}	0.15^{B}	0.19^{A}	0.01	<0.001
C23:0	0.00^{C}	0.05^{B}	0.36^{A}	0.26^{A}	0.06^{B}	0.07	0.013
Monounsaturated	*10.14*	*11.25*	*10.27*	*10.36*	*10.46*	*0.24*	*0.060*
C16:1c9	0.11^{B}	0.15^{A}	0.14^{A}	0.13^{A}	0.15^{A}	0.01	0.009
C17:1c9	0.03	0.03	0.03	0.03	0.04	0.00	0.078
C18:1c9	9.29	10.27	9.30	9.42	9.53	0.24	0.077
C18:1c11	0.68	0.71	0.66	0.66	0.68	0.02	0.251
Polyunsaturated	*41.34*	*41.68*	*38.93*	*38.92*	*36.22*	*0.30*	*0.299*
C18:2n-6	37.83	36.90	33.93	33.98	31.96	1.97	0.281
C18:3n-3	2.18	2.13	1.89	1.84	1.68	0.16	0.242
C20:2n-6	0.20^{A}	0.06^{B}	0.05^{B}	0.00^{C}	0.05^{B}	0.01	<0.001
C20:4n-6	0.09	0.11	0.10	0.07	0.07	0.01	0.185
C20:5n-3	0.40^{C}	0.82^{B}	1.15^{A}	1.20^{A}	1.04^{A}	0.16	0.027
C22:3n-3	0.27^{C}	0.62^{B}	1.22^{A}	1.267 A	0.65^{B}	0.16	0.004
C22:4n-6	0.21^{A}	0.04^{B}	0.05^{B}	0.070 B	0.06^{B}	0.01	<0.001
C22:5n-6	0.00^{C}	0.07^{A}	0.01^{C}	0.012 C	0.032 B	0.01	<0.001
C22:5n-3	0.05^{B}	0.18^{A}	0.00^{C}	0.000 C	0.000 C	0.01	<0.001
C22:6n-3	0.11^{b}	0.25^{a}	0.19^{a}	0.116 b	0.185 a	0.03	0.034
CLA[2] isomers	*0.00*^{B}	*406.00*^{A}	*340.41*^{A}	*372.34*^{A}	*361.37*^{A}	*12.00*	*<0.001*
CLA_11-13tt	0.00^{C}	2.00^{B}	1.26^{B}	0.718^{B}	3.44^{A}	0.86	<0.001
CLA_10-12tt	0.00^{C}	5.31^{A}	3.47^{B}	2.492^{B}	3.79^{B}	0.50	<0.001
CLA_9-11tt	0.00^{C}	4.72^{B}	3.15^{B}	2.656^{B}	60.97^{A}	1.40	<0.001
CLA_8-10tt	0.00^{D}	59.53^{A}	1.65^{C}	2.221^{C}	24.93^{B}	1.53	0.002
CLA_11-13ct	0.00^{B}	0.00^{B}	0.00^{B}	0.00^{B}	17.44^{A}	0.84	<0.001
CLA_10-12tc	0.00^{C}	163.17^{A}	172.51^{A}	182.469^{A}	111.80^{B}	6.21	<0.001
CLA_9-11ct	0.00^{C}	143.51^{A}	126.55^{B}	145.87^{A}	120.11^{B}	4.58	<0.001
CLA_8-10tc	0.00^{D}	16.02^{C}	30.91^{B}	35.30^{A}	18.28^{C}	2.68	<0.001
Index							
ω-6	38.33	37.18	34.14	34.13	32.18	1.97	0.246
ω-3	3.01^{C}	4.01^{B}	4.45^{A}	4.42^{A}	3.56^{B}	0.20	0.002
ω-6/ω-3	12.74^{A}	9.28^{B}	7.89^{C}	7.74^{C}	9.06^{B}	0.71	0.003
Organic acid	*0.59*^{C}	*12.44*^{B}	*28.24*^{A}	*23.79*^{A}	*22.76*^{A}	*4.23*	*<0.001*
lactate	0.13^{B}	3.16^{A}	2.75^{A}	2.39^{A}	3.12^{A}	0.36	<0.001
acetate	0.11^{C}	0.94^{B}	2.01^{A}	1.16^{B}	1.28^{B}	0.14	<0.001
propionate	0.36^{C}	6.83^{B}	21.36^{A}	18.00^{A}	15.91^{A}	3.55	<0.001
butyrate	0.00^{C}	1.51^{B}	2.13^{A}	2.24^{A}	2.45^{A}	0.31	<0.001

[1]SE: Standard Error; [2]CLA: Conjugated Linoleic Acid.

$^{a-c}$: means within rows with different letters significantly differ (P ≤ 0.05)

$^{A-D}$: means within rows with different letters significantly differ (P ≤ 0.01)

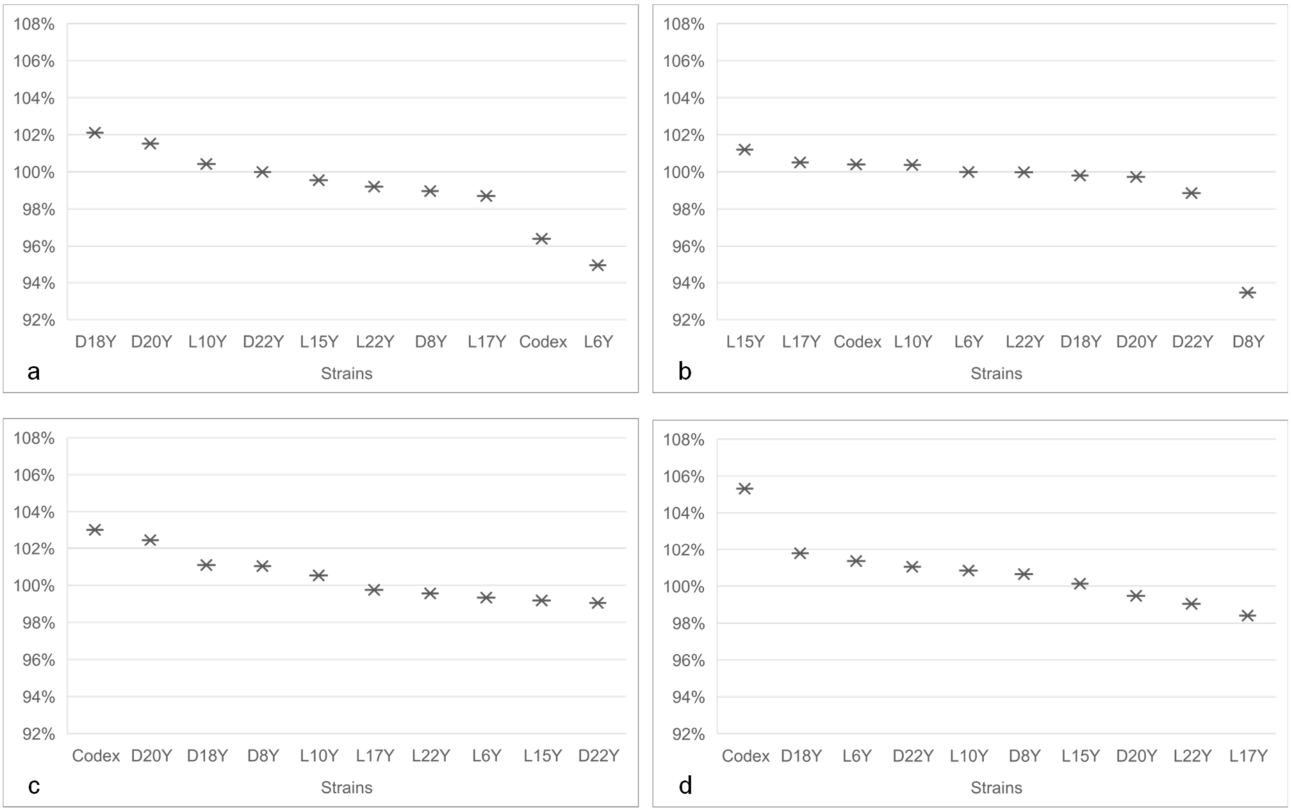

Supplementary Figure S1. Survival percentage of the 10 *Saccharomyces cerevisiae* strains after incubation under simulated gastric conditions at pH 2.0 (**a**), 3.0 (**b**), 8.0 (**c**), and 2.0, with reconstituted skim milk added (10%) (**d**), for 180 min, and further intestinal digestion at pH 8.0 for 360 min. Stars are the mean values (n =3)
